# Phase Composition, Structure and Properties of the Spark Plasma Sintered Ceramics Obtained from the Al_12_Mg_17_-B-Si Powder Mixtures

**DOI:** 10.3390/nano12111895

**Published:** 2022-06-01

**Authors:** Pavel Nikitin, Ilya Zhukov, Alexey Matveev, Sergei Sokolov, Victor Sachkov, Alexander Vorozhtsov

**Affiliations:** Laboratory of Metallurgy Nanotechnologies, National Research Tomsk State University, Lenin Avenue, 36, 634050 Tomsk, Russia; gofra930@gmail.com (I.Z.); alekey.9595@mail.ru (A.M.); sokolovsd95@gmail.com (S.S.); itc@spti.tsu.ru (V.S.); abv1953@mail.ru (A.V.)

**Keywords:** composite materials, B_12_, silicon, spark plasma sintering, phase composition

## Abstract

In this work, composite materials were obtained by spark plasma sintering of an Al_12_Mg_17_-B-Si powder mixture. The structure, phase composition, and mechanical properties of the obtained composites were studied. It was found that various compounds based on B_12_ icosahedrons, such as AlB_12_, B_4_Si, and B_6_Si, are formed during spark plasma sintering. Based on the SEM images and results of XRD analysis of the obtained specimens, a probable scheme for the formation of the phase composition of composite materials during spark plasma sintering was proposed. An increase in the Al_12_Mg_17_-B powder content in the initial mixture from 30 to 70 wt% leads to an increase in hardness from 16.55 to 21.24 GPa and a decrease in the friction coefficient and wear rate from 0.56 to 0.32 and 13.60 to 5.60 10^−5^ mm^−3^/(N/m), respectively.

## 1. Introduction

To date, borides are widely used in the strengthening of traditional materials and the development of new ones, since they demonstrate excellent hardness, corrosion and abrasion resistance, and a high melting point. A wide class of borides is occupied by transition metal diborides. The most common material in this class is titanium diboride TiB_2_, which is reported to have a hardness of up to 35 GPa and an extremely high melting point (3225 °C) that classifies titanium diboride as ultra-high temperature ceramics (UHTCs) [1,2]. Despite their combination of physical and mechanical properties, transition metal diborides have a significantly higher density than higher borides (although their properties are slightly higher): for example, 4.5 g/cm^3^ for TiB_2_ vs 2.58 g/cm^3^ for AlB_12_ [1,3]. Higher borides belong to the group of compounds based on B_12_ icosahedrons. The main compounds based on B_12_ icosahedrons are AlB_12_, B_12_C_3_ (B_4_C), B_12_Si_3_, B_12_Si_2_ (B_4_Si and B_6_Si), and B_12_O_2_ (B_6_O) [3,4,5,6,7,8]. These compounds exhibit excellent properties at relatively low densities, have extremely high melting points due to the strong bonds of intra- and interclusters B_12_ [4]. Thus, in [5], the obtained higher aluminum dodecaborides (AlB_12_) demonstrated excellent hardness from 20 to 30 GPa, and in combination with titanium carbide, the hardness of the AlB_12_ + 20% TiC composite reached 40 GPa at indentation fracture resistance of 5.2 MPa m^1/2^. In [6], the tribological properties of higher borides AlB_12_ and SiB_6_ obtained by spark plasma sintering (SPS) were studied. The samples were found to have a friction coefficient below 0.2 when sliding against Si_3_N_4_, Al_2_O_3_, and SiC. In the last two decades, polycrystalline materials based on AlMgB_14_ have been actively studied [9,10,11]. AlMgB_14_ is also based on B_12_ icosahedrons forming an orthorhombic structure, has high hardness (up to 32 GPa), and a low friction coefficient (COF, 0.08–0.02). The study of AlMgB_14_ + 30 wt% Si composites has shown that their friction coefficient [12] is 0.2, and the microhardness [13] can reach 35–40 GPa. However, it is important to note that during the sintering of aluminum magnesium boride with Si, the pre-reacted AlMgB_14_ powder was used as the initial material, and not the stoichiometric mixture of the Al-Mg-B system.

In connection with the foregoing, it seems interesting to obtain ceramic materials from a stoichiometric mixture of the Al-Mg-B system and silicon and study the processes of phase formation in these materials during spark plasma sintering. At the same time, the question arises whether the formation of AlMgB_14_ is possible in this case, or will a complex composite system consisting of higher borides be formed? Thus, the purpose of this work is to study the phase composition, structure, and properties of ceramics of the Al-Mg-B-Si system obtained by spark plasma sintering.

## 2. Materials and Methods

### 2.1. Raw Materials

Powders of Al_12_Mg_17_, boron, and silicon were used as raw materials. To obtain a stoichiometric mixture of the Al-Mg-B system, intermetallic Al_12_Mg_17_ and amorphous boron powders were mixed in an atomic ratio of Al_12_Mg_17_:B of 2:14. The obtained mixture was mechanically activated in a planetary mill for 3 h to obtain the Al_12_Mg_17_-B powder. Stainless steel balls were used as grinding bodies. The mass ratio of balls to powder mixture was 3:1. The rotation frequency of the planetary mill was 14 Hz. The obtained mechanically activated Al_12_Mg_17_-B mixture was mixed with silicon powder. The limiting mass ratios of the Al_12_Mg_17_-B to Si were considered: 3:7 (70Si-BAM), 5:5 (50Si-BAM), and 7:3 (30Si-BAM), respectively. The average particle size of the initial powders is shown in Table 1.

### 2.2. Process to Obtain Dense Composite Materials

During the next stage, the obtained powder mixtures were cold-pressed in a 12.8-mm-diameter graphite die. The obtained specimens were spark plasma sintered under 70 MPa of pressure. The modes of spark plasma sintering are shown in Table 2. Mechanical pressure was applied to the graphite die in the first minute of the sintering process and maintained constant throughout the process. The maximum sintering temperature of the specimens was chosen as follows. On the one hand, it corresponded to the end of the shrinkage process of the specimens. On the other hand, the maximum sintering temperature was controlled to prevent the silicon powder from melting. Thus, for the specimen 70Si-BAM with the maximum silicon concentration, the sintering temperature was the lowest. The specimen heating rate was 50 °C/min. When the maximum sintering temperature was reached, the current supply was stopped, and the specimens were cooled. The obtained specimens were removed from the graphite die, cleaned of graphite paper, and polished for further research.

### 2.3. Characterization

To determine the amount of oxygen in the intermetallic Al_12_Mg_17_ powder and amorphous boron powder, a LECO ONH (USA) analyser was used. X-ray diffraction analysis of the obtained specimens was performed using a Shimadzu 6000 diffractometer with CuKα radiation and the PDF-4 (Powder Diffraction File) database. The phase composition was refined using the Rietveld method. In this work, the CASTEP program code [14,15] was used to calculate the energies of the reference and refined crystal lattices within the framework of the density functional theory (DFT) using the generalized-gradient approximation (GGA). The microstructure of the Al_12_Mg_17_-B powder and sintered specimens (as well as the mean diameter) was determined using a JEOL JSM-6490 microscope with energy dispersive spectroscopy (EDS). The densities of the sintered specimens were calculated using the Archimedes method. The Vickers hardness (HV) was determined using Metolab-502 microhardness tester at a load of 1 kg (9.8 N). The loading time was 15 s. The friction coefficient and wear rate were studied using a pin-on-disk tribometer in the oscillating mode. The normal load was 5 N, and the speed was 25 mm/s. The sliding distance was 50 m. An Al_2_O_3_ ball was used as a counter-body. The tests were performed under normal conditions at room temperature in air without using a lubricant coating. The macrostructure of the surface of the samples after friction was studied using a Metam LV-34 optical microscope.

## 3. Results and Discussions

### 3.1. Microstructure, Phase Composition and Dispersity of the Al_12_Mg_17_-B Powder

**Figure 1:**
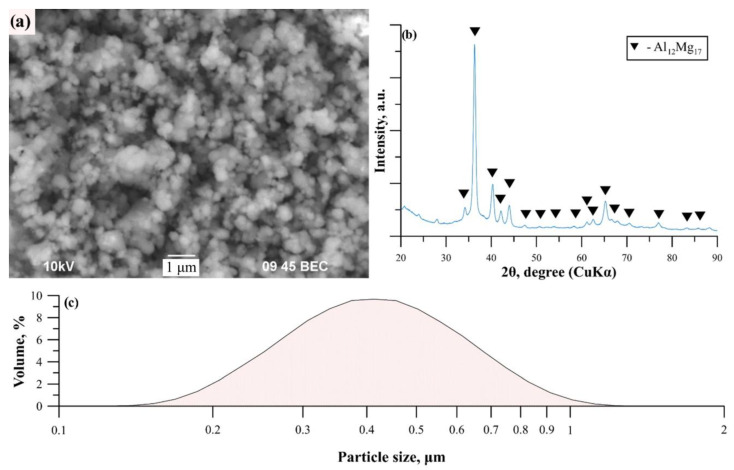
SEM image (**a**), XRD pattern (**b**) and particle size distribution (**c**) of the Al_12_Mg_17_-B powder.

Figure 1 shows the SEM image, XRD pattern, and particle size distribution of the Al_12_Mg_17_-B powder after mechanical activation. As can be seen from Figure 1, the average particle size of the obtained powder mixture is 400 nm.

The results of X-ray structural analysis are shown in Table 3. According to the given XRD pattern, the phase composition of the obtained powder is represented by Al_12_Mg_17_ intermetallic compound, the refined parameters of which differ slightly from the standard. In this case, the halo at small diffraction angles corresponds to the amorphous phase of boron [16]. However, the volume (V) of the refined Al_12_Mg_17_ crystal lattice differs significantly from the reference value as its energy (E) increases, which may indicate deformations of the intermetallic lattice during mechanical activation and its interaction with boron.

### 3.2. Sintering Features, Microstructure and Phase Composition of the Obtained Specimens

**Figure 2:**
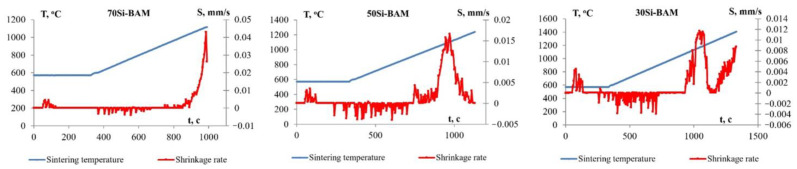
Temperature dependence of shrinkage (L) and shrinkage rate (S) of the Al_12_Mg_17_-B-Si specimens.

Figure 2 shows shrinkage curves for specimens obtained by spark plasma sintering of the Al_12_Mg_17_-B powder and Si. As can be seen from the obtained curves, at a heating rate V_h_ of 50 °C/min, intense shrinkage of the Al_12_Mg_17_-B-Si powder mixtures is observed in the temperature range from 750 to 1350 °C. The maximum shrinkage rate of the 70Si-BAM specimen is observed at a temperature of 1000 °C and is approximately 0.04 mm/s. For the 50Si-BAM system, the maximum shrinkage rate is observed at a temperature of 1000 °C and is approximately 0.016 mm/s, and for the 30Si-BAM system, the maximum shrinkage rate of 0.012 mm/s is achieved at a temperature of 1050 °C. Large concentrations of silicon added to the Al_12_Mg_17_-B powder increase the shrinkage rate of the material and decrease the sintering temperature.

**Figure 3:**
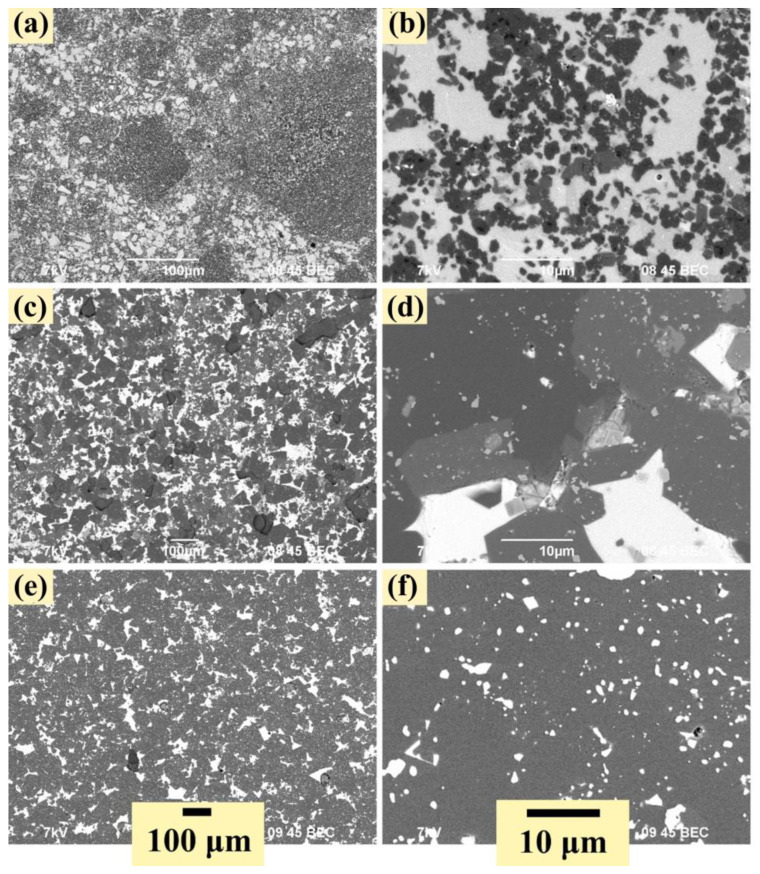
SEM images (BEC) of the spark plasma sintered specimens: (**a**,**b**)—70Si-BAM, (**c**,**d**)—50Si-BAM, (**e**,**f**)—30Si-BAM.

The microstructure of the sintered specimens is shown in Figure 3. Spark plasma sintering of Al_12_Mg_17_-B-Si powder systems leads to the formation of a dense homogeneous structure with a uniform distribution of components in the structure. Moreover, no porosity is observed in the structure of the obtained specimens. The 70Si-BAM specimen (Figure 3a,b) has a structure in which boride compounds (dark areas) are surrounded by silicon particles and agglomerates (light areas). The maximum size of agglomerates does not exceed 40 µm. In this case, the structure of the obtained sample is represented by a large number of submicron particles of both silicon and boride compounds mixed with each other. At the same time, in some areas, boride compounds are interspersed with islands enriched in silicon (Figure 3b). With an increase in the Al_12_Mg_17_-B content to 50 wt.% (specimen 50Si-BAM), the structure of the composite changes. It is represented by separate large agglomerates of boride compounds and silicon (Figure 3c,d). On the contrary, in the microstructure of the 30Si-BAM specimen, silicon agglomerates interspersed in the boron-enriched matrix (Figure 3e,f). The size of silicon agglomerates does not exceed 20 μm. In addition, as can be seen from Figure 3f, there is a large number of submicron silicon particles evenly distributed in the boron-enriched matrix. The results of elemental analysis of the microstructure of the obtained specimens showed that light areas correspond to silicon, while elements of aluminum, magnesium, and boron were found in dark areas, which corresponds to boron-enriched compounds. Oxygen was also found in the structure of the samples. According to the results obtained on the light element analyzer LECO ONH, the amount of oxygen in the raw boron powder is 1.1 wt%, and in the intermetallic Al_12_Mg_17_ powder does not exceed 0.1 wt%. Thus, the presence of oxygen in the structure is due to the contamination of the initial boron powder with boron oxide B_2_O_3_ [17,18].

**Figure 4:**
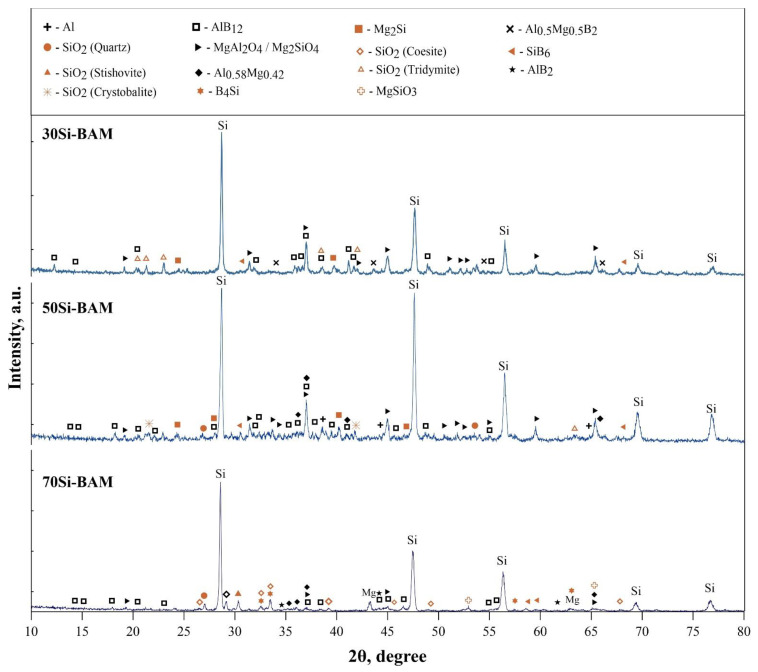
XRD pattern of the obtained specimens.

XRD patterns of the specimens obtained by spark plasma sintering of the Al_12_Mg_17_-B-Si powders are shown in Figure 4. According to the obtained XRD patterns, aluminum dodecaboride AlB_12_ is formed in all specimens, regardless of the silicon concentration. The presence of oxygen in the initial boron powder leads to the formation of a large amount of SiO_2_ oxide ceramics and MgAl_2_O_4_ and Mg_2_SiO_4_ spinels [19,20]. Thus, based on the results of XRD analysis, a probable scheme for the formation of the phase composition of composite materials during spark plasma sintering of the Al_12_Mg_17_-B powder and silicon is proposed. During spark plasma sintering of the 70Si-BAM specimen (sintering temperature of 1115 °C), boron oxide B_2_O_3_ melts and reacts with silicon, aluminum, and magnesium, forming MgAl_2_O_4_/Mg_2_SiO_4_ spinels and silicon oxide modifications. In particular, a coesite phase was found in this specimen, which forms at temperatures up to 1000 °C [21]. In turn, boron reacts with aluminum to form the aluminum dodecaboride (AlB_12_) phase and silicon to form boron silicide B_4_Si (also based on B_12_ icosahedrons). Due to the high concentration of silicon in the specimen, an excess of magnesium was also found, which did not react with other components. With an increase in Al_12_Mg_17_-B concentration up to 50 wt% and sintering temperatures up to 1240 °C, there is a significant change in the phase composition. A cristobalite phase was found in the 50Si-BAM specimen, which forms in the temperature range from 872 to 1725 °C [21]. At the same time, the AlB_12_ concentration in the specimen increases, and due to the high sintering temperature and a decrease in the amount of silicon, boron silicide B_4_Si decomposes into SiB_6_ and silicon [22], which subsequently interacts with magnesium and forms the Mg_2_Si phase. In the 30Si-BAM specimen obtained at a sintering temperature of 1410 °C, a modification of metastable tridymite is formed and the concentrations of other SiO_2_ modifications are significantly reduced. Apparently, this is due to the high sintering temperature of the sample and the insufficient concentration of silicon for its interaction with other elements. It is also important to note that in addition to the AlB_12_ phase, the Al_0.5_Mg_0.5_B_2_ phase is formed in the obtained specimens. In [11,17,23], it was found that the Al_0.5_Mg_0.5_B_2_ diboride phase is an intermediate phase in the formation of aluminum magnesium boride AlMgB_14_. However, the presence of an inert diluent (silicon) absorbs part of the heat energy and prevents the formation of the AlMgB_14_ phase under the selected sintering mode, despite the completion of the specimen consolidation process.

### 3.3. Mechanical Properties of the Sintered Specimens

**Figure 5:**
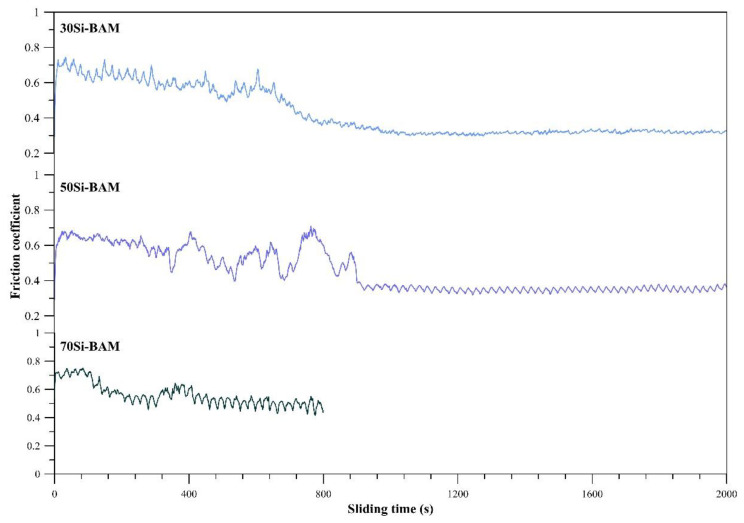
Variation of the friction coefficient of the obtained specimens.

Figure 5 depicts the variations in friction coefficients of the spark plasma sintered specimens. The results of measuring the friction coefficient and wear rate of the obtained specimens are shown in Table 4. As can be seen from the obtained results, an increase in the Al_12_Mg_17_-B powder content in the initial mixture leads to a decrease in the friction coefficient and wear rate of the sintered specimens due to the formation of a larger amount of ceramic particles evenly distributed in the structure. In the process of measuring the friction coefficient of the 70Si-BAM specimen, silicon particles were intensively pulled out from the surface; therefore, the duration of the test was limited to 800 s. The lowest friction coefficient and wear rate of 0.32 and 5.60 10^−5^ mm^−3^/(N/m), respectively, are achieved for the 30Si-BAM specimen with the lowest silicon content. In this case, the friction coefficient smoothly decreases from 0.75 to 0.4 at 800 s of testing and then is fixed at a value of 0.32.

The friction coefficient curve of the 50Si-BAM specimen is represented by several extrema following one after another from 400 to 900 s of testing, after which the friction coefficient decreases and reaches a value of 0.36. The obtained results indicate the release of ceramic particles on the surface of the specimen, which lubricate it and reduce the wear rate to 9.40 10^−5^ mm^−3^/(N/m), and are in good agreement with the reported data [6], where during friction tests of pure AlB_12_ and SiB_6_ specimens, the friction coefficient reached values of 0.15 against Al_2_O_3_ the counter-body. Moreover, the friction coefficient of the obtained samples is also in good agreement with the friction coefficient of SiC-TiB_2_ composite ceramics, which is approximately 0.35 [24].

**Figure 6:**
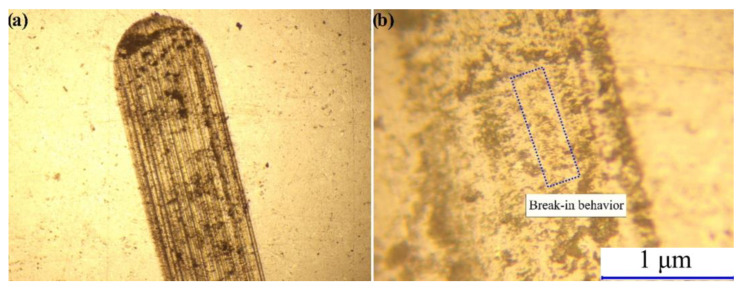
Optical images of the 70Si-BAM surface.

On the contrary, the 70Si-BAM specimen has the highest friction coefficient and wear rate of 0.56 and 13.60 10^−5^ mm^−3^/(N/m), respectively, which is primarily due to the insufficient concentration of wear-resistant ceramic particles in the specimen. Figure 6 shows optical images of the surface of the 70Si-BAM specimen after friction testing. As can be seen from Figure 6, after testing, the surface of the specimen is significantly deformed, while typical break-in behavior is observed in the track area [25], which leads to a large degree of wear of the specimen.

Table 5 shows the values of the density and microhardness of the obtained specimens. It was found that with an increase in the Al_12_Mg_17_-B powder content in the initial powder mixture, the hardness of sintered materials increases. This is due to the formation of compounds based on B_12_ icosahedrons (AlB_12_, B_4_Si, B_6_Si) with high hardness [3,4,5]. At a content of 70 wt% of Al_12_Mg_17_-B powder in the initial mixture, the hardness of the composite reaches 21.24 ± 1.22 GPa. Thus, despite the formation of a large number of oxide phases with lower hardness, icosahedral compounds are dominant in the specimens, which is confirmed by SEM images of sintered specimens (Figure 3).

## 4. Conclusions

In this work, composite materials from the Al_12_Mg_17_-B and Si powders were obtained by spark plasma sintering. The mechanical properties, structure, and phase composition of the obtained materials were studied. Based on the SEM images and results of XRD analysis, a probable scheme for the formation of the phase composition of composite materials was proposed. It was found that icosahedral compounds AlB_12_, B_4_Si, and B_6_Si are formed during spark plasma sintering. The ternary boride AlMgB_14_ is not formed in the obtained specimens, but at a content of the Al_12_Mg_17_-B powder of 70 wt% in the initial mixture, its intermediate phase Al_0.5_Mg_0.5_B_2_ is formed. An increase in the Al_12_Mg_17_-B powder content in the initial mixture from 30 to 70 wt% leads to an increase in hardness from 16.55 ± 0.8 to 21.24 ± 1.22 GPa and a decrease in the friction coefficient and wear rate from 0.56 to 0.32 and 13.60 to 5.60 10^−5^ mm^−3^/(N/m), respectively.

## Figures and Tables

**Table 1:** Characteristics of the raw powders.

Powder	Average Particle Size	Purity, %
Al_12_Mg_17_	15 μm	≥99.2
Amorphous B	600 nm	≥98.7
MA-Al_12_Mg_17_-B	400 nm	≥98.8
Si (OJSC «Polema»)	60 μm	≥99.2

**Table 2:** Designation of specimens and parameters of spark plasma sintering.

Designation	Composition (wt.%)	T_sintering_, °C	V_heating_, °C/min	P, MPa
70Si-BAM	70% Si + 30% Al_12_Mg_17_-B	1115	50	70
50Si-BAM	50% Si + 50% Al_12_Mg_17_-B	1240	50	
30Si-BAM	30% Si + 70% Al_12_Mg_17_-B	1410	50	

**Table 3:** Structural parameters of the detected phase in the Al_12_Mg_17_-B powder.

Phase	State	a, Å	α	V, Å3	E, eV
Al_12_Mg_17_	Reference	10.549	90	1174.0	−34,471.7
	Refined	10.405	90	1126.4	−34,469.3

**Table 4:** Friction coefficient and wear rate of the sintered specimens.

Designation	T_sintering_, °C	Friction Coefficient	Wear Rate, 10^−5^ mm^−3^/(N/m)
70Si-BAM	1115	0.56	13.60
50Si-BAM	1240	0.36	9.40
30Si-BAM	1410	0.32	5.60

**Table 5:** Properties of the sintered specimens.

Designation	Density, g/cm^3^	Hardness (HV), GPa
70Si-BAM	2.37 ± 0.01	16.55 ± 0.83
50Si-BAM	2.16 ± 0.04	18.4 ± 1.35
30Si-BAM	2.30 ± 0.01	21.24 ± 1.22

## Data Availability

The data presented in this study are available in the article.
